# Editorial: Lung Ultrasound and COVID-19 Interstitial Pneumonia in Different Medical Care Settings

**DOI:** 10.3389/fmed.2022.951706

**Published:** 2022-06-23

**Authors:** Luigi Vetrugno, Lorenzo De Marchi, Belaid Bouhemad

**Affiliations:** ^1^Department of Medical, Oral and Biotechnological Sciences, University of Chieti-Pescara, Chieti, Italy; ^2^Department of Anesthesiology, Critical Care Medicine and Emergency, SS. Annunziata Hospital, Chieti, Italy; ^3^Department of Anesthesiology, MedStar-Georgetown University Hospital, Washington, DC, United States; ^4^Department of Anesthesiology and Intensive Care, C.H.U. Dijon, Dijon, France

**Keywords:** lung ultrasound, COVID-19, outpatient, pneumonia, SARS-CoV-2

The last decade has been characterized by the widespread use of lung ultrasound (LUS), initially in the emergency department and subsequently in the intensive care unit (1, 2). After the beginning of the SARS-CoV2 pandemic, LUS use has exponentially increased worldwide beyond these two clinical areas (3). With this editorial, we speculate on the primary reasons for such growth. First, in patients that developed adult respiratory distress syndrome (ARDS) secondary to COVID-19 pneumonia, LUS findings are comparable with computed tomography (CT) scan (3). Second, compared with other diagnostic devices, lung ultrasound can be utilized as a low-cost point-of-care device that allows diagnosis and management of COVID-19 patients at home. Third, the reproducibility of LUS makes it an ideal monitoring tool that can help follow the course of the lung disease (4). For these reasons, skepticism toward the technique progressively decreased during the pandemic, while the number of providers utilizing LUS as a diagnostic tool increased.

In this special issue, “Lung Ultrasound and COVID-19 Interstitial Pneumonia in Different Medical Care setting,” the first point is addressed by Vetrugno et al. in their article on the use of LUS in COVID-19 pregnant women. As reported in the article, pregnant women carry a three times higher risk of developing severe COVID-19 pneumonia from SARS-CoV-2 infection. The authors describe how LUS can reduce the number of chest radiographs and CT scans in this patient population (Vetrugno et al.). In this study, the most frequent pathological LUS findings are focal B lines and the “light beam” sign (Vetrugno et al.). Since LUS score significantly correlates with patients' clinical symptoms, this tool can identify patients at higher risk for admission to the intensive care unit. The authors also report that the time needed to perform the LUS exam was very short (<5 min) despite having to wear bulky personal protective equipment (5).

A second article by Chevallier Lugon et al. shows that lung ultrasound helps preserve local health system resources. The authors propose some algorithms to select patients who are candidates for outpatient follow-up rather than requiring hospitalization. In the study, LUS performed with portable devices outside of the hospital setting help reach isolated patients at home during lockdown periods. Furthermore, the authors show a good association between clinical findings, LUS abnormalities, and chest radiograph results (Chevallier Lugon et al.). They suggest that LUS may replace standard chest radiographs, limiting patients' movement and risk of contamination by SARS-CoV-2. A close follow-up is needed for patients with COVID-19 who could develop interstitial lung disease (ILD), and pulmonary fibrosis (PF). In these patients, Clofent et al. show the superiority of LUS over chest radiographs and an equal sensitivity and negative predictive value compared to CT for detecting connective tissue disease-associated ILD. LUS can be used as a first-line approach to rule out post-COVID-19 lung sequelae through findings like B lines, potentially reducing the use of high-resolution CT, and improving the efficiency of health care services.

Finally, this Frontier in Medicine special issue includes a final article describing the risk of neuraxial anesthesia-related hypotension in COVID-19 parturients undergoing cesarean delivery. The findings of from Zhang et al. show an increased risk of neuraxial anesthesia-related hypotension in COVID-19 parturients with a prevalence of neuraxial anesthesia-related hypotension higher in this patients population compared to healthy. The incidence of autonomic dysfunction results to be equal to 57.4% in COVID-19 parturients compared and 41.9% in the control group (Zhang et al.).

In conclusion, COVID-19 has transformed our daily clinical practice forever as shown by the example of LUS use. However, despite all the popularity that LUS gained during this pandemic, we have to remember that B-lines, like other LUS signs, have poor specificity. Therefore, LUS results must always be interpreted in a clinical context like many other imaging tests. For this reason, we advocate for better and more accessible training, along with evidence-based guidelines regarding the use of LUS as a diagnostic tool. This can only be achieved in the future years through the implementation of lecture series, seminars, and a certification course (6).

## Author Contributions

LV, LD, and BB conceived the editorial and wrote the text. LD critically revised the manuscript and coordinated LV and BB work. All authors contributed to the article and approved the submitted version.

## Conflict of Interest

The authors declare that the research was conducted in the absence of any commercial or financial relationships that could be construed as a potential conflict of interest.

## Publisher's Note

All claims expressed in this article are solely those of the authors and do not necessarily represent those of their affiliated organizations, or those of the publisher, the editors and the reviewers. Any product that may be evaluated in this article, or claim that may be made by its manufacturer, is not guaranteed or endorsed by the publisher.
